# Comparative analysis of three different protocols for cholinergic neuron differentiation in vitro using mesenchymal stem cells from human dental pulp

**DOI:** 10.1080/19768354.2019.1626280

**Published:** 2019-06-10

**Authors:** Young-Hoon Kang, Sharath Belame Shivakumar, Young-Bum Son, Dinesh Bharti, Si-Jung Jang, Kang-Sun Heo, Won-Uk Park, June-Ho Byun, Bong-Wook Park, Gyu-Jin Rho

**Affiliations:** a Department of Dentistry, Gyeongsang National University School of Medicine and Institute of Health Science, Jinju, Republic of Korea; b Department of Oral and Maxillofacial Surgery, Changwon Gyeongsang National University Hospital, Changwon, Republic of Korea; c Department of Theriogenology and Biotechnology, College of Veterinary Medicine and Research Institute of Life Science, Gyeongsang National University, Jinju, Republic of Korea; d Department of Dental Technology, Jinju Health College, Jinju, Republic of Korea

**Keywords:** Cholinergic neurons, in vitro differentiation, dental pulp stem cells, mesenchymal stem cells, acetylcholine

## Abstract

A decrease in the activity of choline acetyltransferase, the enzyme responsible for acetylcholine synthesis in the cholinergic neurons cause neurological disorders involving a decline in cognitive abilities, such as Alzheimer’s disease. Mesenchymal stem cells (MSCs) can be used as an efficient therapeutic agents due to their neuronal differentiation potential. Different source derived MSCs may have different differentiation potential under different inductions. Various in vitro protocols have been developed to differentiate MSCs into specific neurons but the comparative effect of different protocols utilizing same source derived MSCs, is not known. To address this issue, dental pulp derived MSCs (DPSCs) were differentiated into cholinergic neurons using three different protocols. In protocol I, DPSCs were pre-induced with serum-free ADMEM containing 1 mM of β-mercaptoethanol for 24 h and then incubated with 100 ng/ml nerve growth factor (NGF) for 6 days. Under protocol II, DPSCs were cultured in serum-free ADMEM containing 15 µg/ml of D609 (tricyclodecan-9-yl-xanthogenate) for 4 days. Under protocol III, the DPSCs were cultured in serum-free ADMEM containing 10 ng/ml of basic fibroblast growth factor (bFGF), 50 µM of forskolin, 250 ng/ml of sonic hedgehog (SHH), and 0.5 µM of retinoic acid (RA) for 7 days. The DPSCs were successfully trans-differentiated under all the protocols, exhibited neuron-like morphologies with upregulated cholinergic neuron-specific markers such as ChAT, HB9, ISL1, BETA-3, and MAP2 both at mRNA and protein levels in comparison to untreated cells. However, protocol III-induced cells showed the highest expression of the cholinergic markers and secreted the highest level of acetylcholine.

## Introduction

Neurons are the basic functional units of the nervous system facilitating the transmission of electrical impulses. MSCs when treated with neuron specific inductions can result into the development of functionally distinct types of neurons such as cholinergic neurons, amino acidergic neurons (γ-aminobutyrinergic neurons), and aminergic neurons (dopaminergic, 5-hydroxytryptaminergic, and norepinephrinergic neurons) (Sun et al. 2013). These different types of neurons have different capabilities to release specific neurotransmitters and play specialized roles. Any deformities in the functioning of these neurons results into neurological disorders. Therefore, strategies concerning the protection or development of these valuable cells are very much needed to overcome such problems which can be made possible by using cell based therapies. In one such example of a pathological condition in a monkey model of Huntington’s disease, degeneration of amino acidergic neurons has been lowered by using encapsulated cells delivering neuroprotective effects by producing neurotrophic factor CNTF (Cilliary neurotrophic factor) (Emerich et al. 1997). Similarly, aminergic neurons found in the basal ganglia and the lower brain stem are reportedly associated with Parkinson’s disease (Kones 2010), regulation of the sleep-wakefulness cycle, and sleep disorders (Szabadi 2006). The cholinergic neurons play pivotal roles in cognition, attention, learning, and behavioural responses (Luchicchi et al. 2014). The loss or dysfunction of cholinergic neurons leads to the reduction of acetylcholine (Ach) resulting in the motor nerve degeneration and irreversible cognitive decline as observed in Alzheimer’s disease (Lee et al. 2010). Although different approaches have been used for regenerating the cholinergic neurons, none of them has been effective enough to be therapeutically used. And the comparative effect of available differentiation strategies using the same source derived MSCs has not been much elucidated. Also, there is an immense need to understand the effect and efficacy of the available protocols along with the development of new safer and highly efficient strategies with a special focus on utilization of MSCs as therapeutic agents. Therefore, stem cell-based therapy has recently gained much attention and holds great promise because of its translational relevance (Kim et al. 2009; Lindvall et al. 2004).

Recent studies demonstrated the in vitro and in vivo differentiation potential of the mesenchymal stem cells (MSCs) from various sources into distinct nerve cells, including Schwann cells, dopaminergic and cholinergic neurons (Chen et al. 2007; Gartner et al. 2014; Hei et al. 2016; Marei et al. 2018). MSCs derived from the dental pulp exhibit potent neurogenic differentiation capacity (Chang et al. 2014; Hei et al. 2016). Dental pulp tissue originates from the neural crest and can be easily obtained from the extracted wisdom teeth and preserved long-term with the newly developed cryopreservation techniques (Huang et al. 2009; Ullah et al. 2017). Considering the origin of DPSCs and their specificity towards neuronal differentiation, three different protocols were used to evaluate the expression level of neuronal markers as well as their functional ability under different induction conditions. Moreover, it could be more valuable to check the in vitro differentiation potential of the same source derived MSCs under different induction conditions so that cell behaviour under different treatments along with a selection of best treatments can be made. Our previous studies demonstrated the MSC characteristics in the dental pulp-derived stem cells (DPSCs) and their multi-lineage differentiation potential including a successful in vitro differentiation into cholinergic neurons. In addition, in vivo transplantation of DPSCs or the differentiated cholinergic neurons (dChN) improved the motor nerve regeneration and behavioural activities in animals with peripheral nerve defects (Ullah et al. 2017; Jang et al. 2018). Previously, it has been demonstrated that the same source derived MSCs show positive but varied differentiation potential under different induction conditions (Verpelli et al. 2013; Marei et al. 2018).

Different chemical signalling molecules such as cytokines and growth factors were employed to differentiate the cholinergic neurons from pluripotent stem cells. Our intense literature survey revealed three different protocols for the in vitro cholinergic neuron differentiation from bone marrow MSCs. The first protocol (Protocol I) utilizes β-mercaptoethanol (BME) and nerve growth factor (NGF) in cholinergic induction media (Naghdi et al. 2009a, 2009b). The second protocol (Protocol II) involved the addition of tricyclodecane-9-yl-xanthogenate (D609) in their induction media, upon which, Ach secreted by the cholinergic neurons was detected indicating differentiation (Wang et al. 2004; Wang et al. 2007; Sun et al. 2013; Jang et al. 2018). The third protocol (Protocol III) employed basic fibroblast growth factor (bFGF), sonic hedgehog (SHH), and retinoic acid (RA) in the culture media, which resulted in improved differentiation of the motor neurons (Goncalves et al. 2009; Qi et al. 2010). Therefore, the comparative effect of cytokines, signalling molecules and phosphatidylcholine-specific phospholipase C inhibitor was evaluated under different induction conditions.

The main purpose of this study was to compare the efficacy of these protocols on the basis of the differentiation potential of the DPSCs. The extracted wisdom teeth were used as a source of DPSCs. Their MSC properties and the multipotent characteristics were analysed and then differentiated into cholinergic neurons in vitro using three different induction protocols. The cholinergic neuronal differentiation potential was compared based on the morphological changes, expression of cholinergic neuron-specific markers, and secretion of acetylcholine.

## Materials and methods

### Chemicals and media

All chemicals were purchased from Sigma-Aldrich (St. Louis, MO, USA) and the culture media were procured from Gibco (Invitrogen, Grans Island, NY, USA) unless otherwise specified. The pH of the media was adjusted to 7.4 and the osmolality was adjusted to 280 mOsm/kg.

### Isolation, morphological assessment and proliferation rate of DPSCs

Human dental pulp tissues were collected in sterile containers containing Dulbecco’s phosphate-buffered saline (DPBS) after obtaining the informed consent under the medical guidelines set by the GNUH IRB-2012-09-004. Dental pulps were harvested from the extracted wisdom teeth (*n* = 4; 2 males and 2 females; aged 15–19 years) at the Department of Oral and Maxillofacial Surgery, Changwon Gyeongsang National University Hospital. Isolation was performed as per the previously published protocol (Ullah et al. 2017). Briefly, tissues were minced into 1–3 mm^2^ explants and digested with 1 mg/ml collagenase type I for 40 min at 37°C. Enzyme inactivation was done with advanced Dulbecco’s modified Eagle’s medium (ADMEM) containing 30% FBS followed by sequential filtration using a 100 and 40 µm nylon cell strainer (BD Falcon, Bedford, MA, USA) and centrifugation at 500 × g for 5 min. Cell pellet was reconstituted in 10% ADMEM and cultured in a humidified incubator under 5% CO_2_ at 37°C. At 70%–80% confluence, the cells were dissociated with 0.25% (w/v) trypsin-EDTA solution and subcultured till passage 3. The cells from passage 3 were used in all the experiments for further characterization and analysis unless specified otherwise.

Morphological assessments were made under a light microscope and images were taken at 100x magnification using DIAPHOT 300 microscope (Nikon, Tokyo, Japan). DPSC from the three different dental sources were used to evaluate the proliferation rate using MTT [3-(4, 5-dimethylthiazol-2yl)-2, 5-diphenyltetrazolium bromide] assay. Briefly, DPSCs were seeded at a density of 9 × 10^3^ cells/well in 10% ADMEM on a 24-well culture plate. Three independent experiments were performed, each in biological triplicates. After the specified time intervals, MTT (1 mg/ml) (Sigma-Aldrich) was added to each well at specific time points (24, 48, 72 h, & 96 h) and incubated at 37°C for 4 h. The culture media were then removed and the DPSCs were washed twice with DPBS. The insoluble formazan crystals formed upon MTT reduction by viable cells was dissolved in dimethyl sulfoxide (Sigma-Aldrich) and the absorbance was read at 570 nm using a plate reader.

### Characterization of DPSCs with flow cytometry and cell cycle analyses

DPSCs were analysed for the expression of cell surface markers (CD markers) and the DNA content using a flow cytometer (BD FACSVerse; Becton Dickinson, NJ, USA) according to previously published protocols (Jang et al. 2018). In brief, the trypsinized DPSCs were fixed using 3.7% paraformaldehyde solution at 20°C for 30 min. The cells (1 × 10^5^ cells) were then washed twice with DPBS and labelled with the respective conjugated antibodies for flow cytometry analyses. The antibodies used in the study are fluorescein isothiocyanate (FITC) Mouse Anti-Human CD34 (1:100; BD Pharmingen, San Jose, CA, USA), FITC Mouse Anti-Human CD45 (1:100; BD Pharmingen), FITC Mouse Anti-Human CD90 (1:100; BD Pharmingen), FITC Mouse Anti-Human CD73 (1:100; BD Pharmingen), Mouse Anti-Human CD14, (1:100) (1:100; Santa Cruz Biotechnology, Inc., Dallas, TX, USA), Mouse Anti-Human CD19 (1:100; Santa Cruz), Mouse Anti-HLA-DR (1:100; Santa Cruz), Mouse Anti-Human CD105 (1:100; Santa Cruz), and Mouse Anti-Human vimentin (1:100; Sigma-Aldrich). The cells were incubated with the primary antibodies for 30 min under dark. The unconjugated primary antibodies were further incubated with secondary FITC-conjugated goat anti-mouse IgG (1:100; BD Pharmingen) for 30 min under dark. For isotype matched negative control, Mouse IgG1 (1:100; BD Pharmingen) was used. A total of 10,000 labelled cells per sample were acquired and analysed. For the cell cycle analysis, a total of 1 × 10^6^/mL DPSCs at passage 3 were fixed in 70% ethanol at 4°C for 4 h. Subsequently, the cells were stained with 10 µg/ml propidium iodide solution for 15 min after washing cells twice with DPBS and acquired using the flow cytometer. The experiments were repeated three times with biological triplicates each time.

### Evaluation of in vitro mesenchymal lineage differentiation potency of DPSCs

The in vitro differentiation potential of DPSCs (into adipocytes, osteocytes, and chondrocytes) was evaluated using a previously published protocol with minor modifications (Jang et al. 2018). Briefly, adipogenesis was carried out in the adipogenic medium with 10 µM insulin, 1 µM dexamethasone, 100 µM indomethacin, and 500 µM isobutylmethylxanthine (IBMX). Adipogenesis was confirmed by the accumulation of oil droplets upon staining of the differentiated cells with Oil red O solution. Osteogenesis was performed in the osteogenic medium consisting of 50 µM ascorbate-2-phosphate, 0.1 µM dexamethasone, and 10 mM glycerol-2-phosphate. Osteogenesis was then confirmed by the accumulation of calcium nodules upon staining of the differentiated cells with alizarin red and von Kossa. Chondrogenesis was carried out using the commercial chondrogenic medium (StemPro® Osteocyte/Chondrocyte Differentiation Basal Medium; StemPro® Chondrogenesis supplement, Gibco, Life technologies). Chondrogenesis was confirmed by staining the differentiated cells with Safranin O and Alcian blue.

### Cholinergic neuronal differentiation of hDPSCs using three different protocols

In vitro cholinergic neuronal differentiation was performed using three different protocols as reported earlier with minor modifications (Wang et al. 2007; Naghdi et al. 2009a; Qi et al. 2010). Briefly, 5 × 10^4^ cells were grown on each well of pre-coated six-well plates (Thermo Scientific, Roskilde, Denmark) in ADMEM with 10% FBS. After reaching 70%–80% confluence, the cells were cultured in each induction medium. Under protocol I, pre-induction of DPSCs was done with serum-free ADMEM containing 1 mM of BME for 24 h, and subsequently, the cells were incubated with 100 ng/ml of NGF as cholinergic neuron inducer for another 6 d (Naghdi et al. 2009a). Under protocol II, the DPSCs were cultured in serum-free ADMEM containing 15 µg/ml of D609 (Tricyclodecan-9-yl-xanthogenate) for 4 days (Wang et al. 2007). Under protocol III, the DPSCs were cultured in serum-free ADMEM containing 10 ng/ml of bFGF, 50 µM of forskolin, 250 ng/ml of SHH, and 0.5 µM of RA for 7 days (Qi et al. 2010). The changes in morphology were observed under a DIAPHOT 300 phase contrast microscope (Nikon, Tokyo, Japan).

### Analysis of gene expression by Real-time Quantitative PCR (RT-qPCR)

The expression of pluripotent genes was evaluated by PCR. The fold change of the expression of the mesenchymal lineage-specific genes and cholinergic neuronal marker genes were evaluated by RT-qPCR from biological triplicates. The experiment was repeated three times. Total RNA was isolated from DPSCs using easy-spin^TM^ [DNA free] total RNA extraction kit (iNtRON Biotechnology, Burlington, MA, USA). The complementary DNA (cDNA) was synthesized from 2 µg RNA using HiSenScript^TM^ RH [-] RT PreMix Kit (iNtRON Biotechnology). The reaction involved reverse transcription at 42°C for 50 min, followed by enzyme inactivation at 85°C for 10 min. Subsequently, PCR was carried out using a Maxime PCR Premix (iNtRON Biotechnology), 2 µl cDNA, 1 µl each of forward and reverse primers (10 µM), and 16 µl RNase-free water. PCR was performed under the following conditions: initial denaturation at 94°C for 2 min, followed by 35 cycles of denaturation at 94°C for 30 sec, annealing at 55–60°C for 20 sec, and extension at 72°C for 30 sec and the final extension was performed at 72°C for 10 min using Mastercycler® pro (Eppendorf^TM^, Fisher Scientific Co., Ottawa, ON, Canada). The PCR products were run on a 1.5% agarose gel to check for the amplification. Real-time qPCR was performed in Rotor gene Q (Qiagen, Valencia, CA, USA) using RealMOD^TM^ Green AP 5x qPCR mix (iNtRON Biotechnology). Each reaction mix with a final volume of 25 µl consisted of 50 ng cDNA, 5 µl qPCR mix, 13 µl RNase free water and 1 µl each of forward and reverse primers at 400 nM final concentration. The reaction involves initial denaturation at 95°C for 10 min, followed by 40 PCR cycles of 95°C for 10 sec, respective annealing temperature for 6 sec, and 72°C for 4 sec, followed by a melting curve from 60°C to 95°C at 1°C/sec, and then cooling at 40°C for 30 sec, following manufacturer’s instructions. The Ct values and melting curves of each sample were analysed using Rotor-Gene Q series software (Qiagen). YWHAZ (Tyrosine 3-monooxygenase/tryptophan 5-monooxygenase activation protein, zeta polypeptide) was used as the internal control for normalization. The relative mRNA levels of the target genes were calculated by the 2^-ΔΔCT^ method. The specificity of the primers was analysed by their product length as visualized on a 1.5% agarose gel. The primers used in the study are listed in Table 1.

**Table 1.** List of primers used in the study.

Gene	Primer sequence	Product size (bp)	Annealing temp (°C)	Accession no.
*OCT4*	F: AAGCAGCGACTATGCACAAC R: AGTACAGTGCAGTGAAGTGAGG	140	59	NM_002701.5
*SOX2*	F: CACCCACAGCAAATGACAGC R: AGTCCCCCAAAAAGAAGTCCAG	120	59	NM_003106.3
*NANOG*	F: GCAGATGCAAGAACTCTCCAAC R: CTGCGTCACACCATTGCTATTC	133	55	AB093576.1
*PPARγ*	F: TTGCTGTCATTATTCTCAGT R: GAGGACTCAGGGTGGTTCAG	124	60	AB565476.1
*FABP4*	F: TGAGATTTCCTTCATACTGG R: TGGTTGATTTTCCATCCCAT	128	60	NM_001442.2
*LPL*	F: AGACACAGCTGAGGACACTT R: GCACCCAACTCTCATACATT	137	60	NM_000237.2
*RUNX2*	F: ATGTGTGTTTGTTTCAGCAG R: TCCCTAAAGTCACTCGGTAT	199	60	NM_001024630.3
*OSTEONECTIN*	F: GTGCAGAGGAAACCGAAGAG R: AAGTGGCAGGAAGAGTCGAA	202	60	J03040.1
*BMP2*	F: TAGACCTGTATCGCAGGCAC R: GGTTGTTTTCCCACTCGTTT	149	60	NM_001200.2
*SOX9*	F: ATGGAGCAGCGAAATCAACG R: CAAAGTCCAAACAGGCAGAGAG	118	60	BC007951.2
*AGGRECAN*	F: GAATGGGAACCAGCCTATACC R: TCTGTACTTTCCTCTGTTGCTG	98	60	NM_001135.3
*COLLAGEN II*	F: GAGACCTGAAACTCTGCCACC R: TGCTCCACCAGTTCTTCTTGG	165	60	NM_001844.4
*BETA-3*	F: AGTGTGAAAACTGCGACTGC R: ATACTCCTCACGCACCTTGC	110	60	U47634.1
*MAP2*	F: TGGCATTGACCTCCCTAAAGAG R: TTGCTTCCGTTGGCATTTCG	80	60	NM_002374
*HB9*	F: AAACTTGAAACCGCCTCTGG R: AACGCTCGTGACATAATCCC	106	60	NM_001165255.1
*ISL1*	F: TGGTCATTGCCTTGCCAAAC R: TCAAACCAATGCAGCTCCAC	106	60	XM_011543380.2
*CHAT*	F: TCCATTCCCACTGACTGTGCCA R: GATGCTGCTGTTCTGAGCCACC	135	60	NM_001142933.1
*YWHAZ*	F: CTTCACAAGCAGAGAGCAAAG R: CGACAATCCCTTTCTTGTCATC	102	55	NM_003406.3

### Immunocytochemistry (ICC)

The cells under different experimental groups were fixed using 3.7% paraformaldehyde for 30 min and permeabilized with 0.25% Triton X-100 for 10 min at 20°C. The cells were then blocked with 1% BSA for 1 h and after washing the fixed cells twice with DPBS, they were incubated with primary antibodies at 4°C overnight. The primary antibodies used in this study were rabbit anti-ChAT (choline acetyltransferase) (1:100; Abcam), goat anti-HB9 (Homeobox HB9; Motor neuron and pancreas homeobox 1, MNX1) (1:100; Santa Cruz), mouse anti-ISL1 (Insulin gene enhancer protein ISL-1) (1:100; Santa Cruz), mouse anti-BETA-3 (β-3 tubulin) (1:100; Santa Cruz), and goat anti-MAP-2 (microtubule-associated protein 2) (1:100; Santa Cruz). The cells were then incubated with the secondary antibodies for 1 h at 37°C. The secondary antibodies used in this study are CruzFluor^TM^ 488-conjugated goat anti-rabbit IgG (1:100; Santa Cruz), CruzFluor^TM^ 594-conjugated donkey anti-goat IgG (1:100; Santa Cruz), or FITC-conjugated donkey anti-mouse IgG (1:100; Santa Cruz) according to the primary antibody source. The nuclei were counterstained with 1 µg/ml DAPI for 5 min and the images were acquired using a fluorescence microscope (Leica, Germany). The negative controls with only secondary antibodies were used to assess their specificity.

The quantification analyses of ICC for the expressions of the cholinergic neuronal marker proteins were performed by calculating the fluorescence intensity density using ImageJ software (Version 1.52a, National Institute of Health, USA). The integrated density was defined as the sum of the values of each pixel in the image. This is equivalent to the product of the area and the mean grey value. Three independent experiments were performed for each experimental group for the statistical analysis.

### Analysis of acetylcholine (Ach) secretion in culture media

The concentration of Ach in the supernatant of the induction medium after in vitro differentiation of DPSCs into cholinergic neuron-like cells was measured as previously described (Jang et al. 2018). An aliquot of the culture supernatant was collected and the Ach concentration was measured using Amplex® RED Acetylcholine/Acetylcholinesterase Assay kit (Invitrogen) following the manufacturer’s instructions. The Ach was quantified indirectly using 10-acetyl-3,7-dihydroxyphenoxazine (Amplex® Red Reagent), a sensitive fluorogenic probe for H_2_O_2_, an end product of the choline oxidation process. The fluorescence was measured using a fluorescence plate reader at 550 nm for excitation and 595 nm for emission after 1 h of incubation at 20°C with the reaction mix. The number of cells was counted in both the control and the differentiated groups after trypsinization. The concentration of Ach was calculated and presented as µM per 10^5^ cells (µM/10^5^ cells) of culture supernatant.

### Statistical analysis

One-way ANOVA was performed using SPSS 21.0 to determine the statistical differences between the experimental groups and Tukey’s test was conducted for multiple comparisons. Error bars indicate mean ± standard error. Mean values were drawn from the biological triplicates obtained from each of the three independent experiments. *p* < 0.05 was considered statistically significant.

## Results

### Growth characteristics and phenotyping of DPSCs

**Figure 1.**
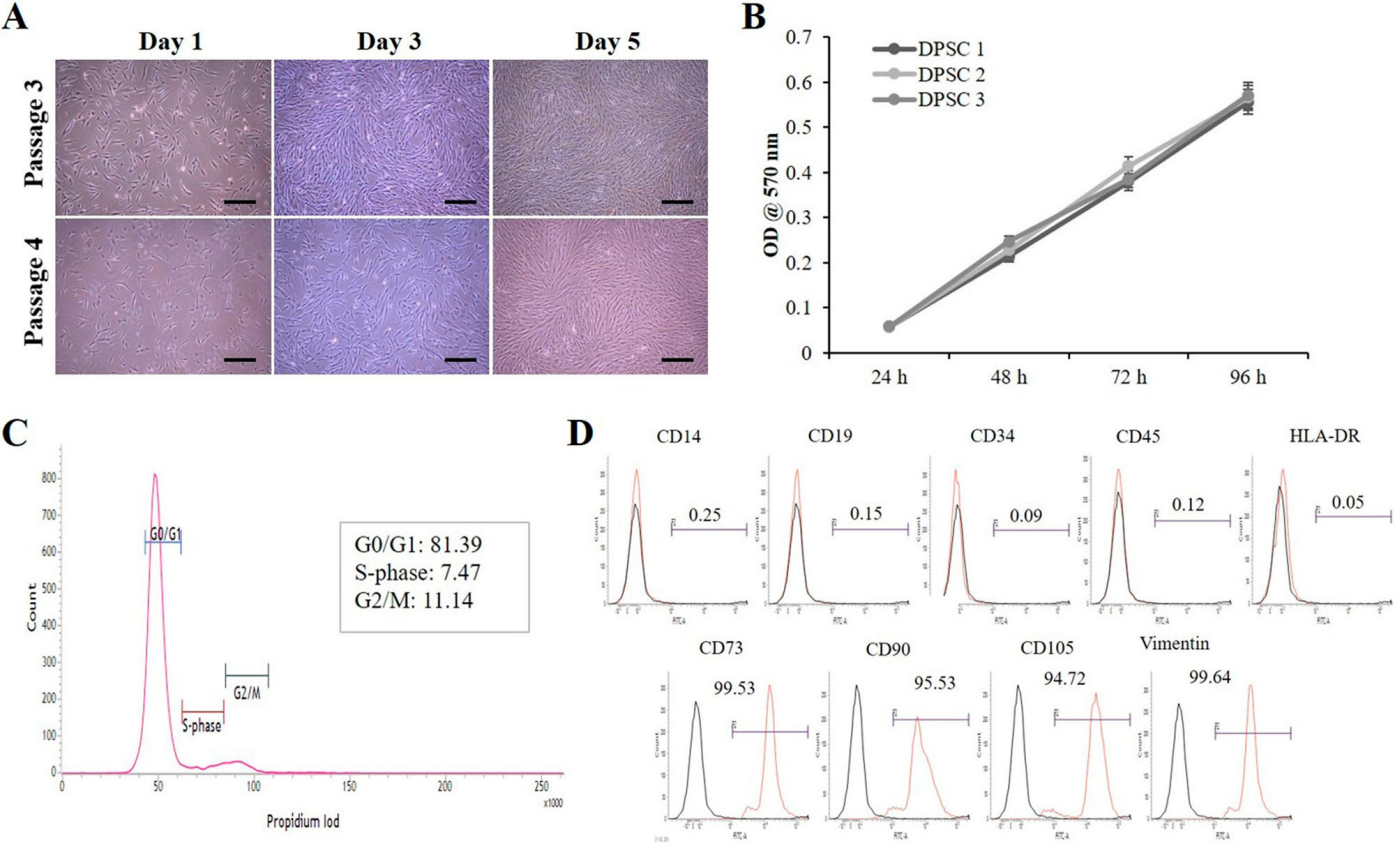
Morphology and growth characteristics of DPSCs. (A) In vitro morphological changes in passage 3 & 4 DPSCs observed under a phase contrast microscope at different culture times showing homogeneous adherent fibroblast-like morphology (Scale bar = 100 µm). (B) Evaluation of cell proliferation using MTT assay. The isolated DPSCs showing normal cell proliferation. Data represent mean ± SEM. (C) Flow cytometry analysis of DPSCs at passage 3 showing normal DNA content in the gap 0/1 (G0/G1), synthesis (S), and gap 2/mitotic (G2/M) phases of the cell cycle. (D) Flow cytometry analysis of DPSCs for cell surface markers. DPSCs at passage 3 were positive for the expression of mesenchymal markers such as CD73, CD90, CD105, and vimentin, and negative for the expression of monocyte marker CD14, B-lymphocyte markers CD19 and HLA-DR, and the hematopoietic markers CD34 and CD45.

After isolation of DPSCs from dental pulp tissues, the cells were cultured in 10% ADMEM and maintained in a humidified incubator with 5% CO_2_. Adherent colonies with fibroblast-like spindle morphology were observed within the first week of culture. These cells became homogeneous at passage 3 upon sub culturing (Figure 1(A)). The number of cells increased with increase in time, indicating an increase in cell proliferation as evaluated by the MTT assay (Figure 1(B)). Our flow cytometry analysis indicated 81.39%, 7.47%, and 11.14% of cells in G0/G1, S, and G2/M phases of the cell cycle, respectively (Figure 1(C)). Further analysis of DPSCs at passage 3 for cell surface markers by flow cytometry revealed positive expression for the MSC-markers such as CD73, CD90, CD105, and vimentin (Figure 1(D)). On the other hand, these cells were negative for the expression of the monocyte marker CD14, B-lymphocyte markers CD19 and HLA-DR, and the hematopoietic markers CD34 and CD45 (Figure 1(D)).

### In vitro differentiation of DPSCs into mesenchymal lineages

**Figure 2.**
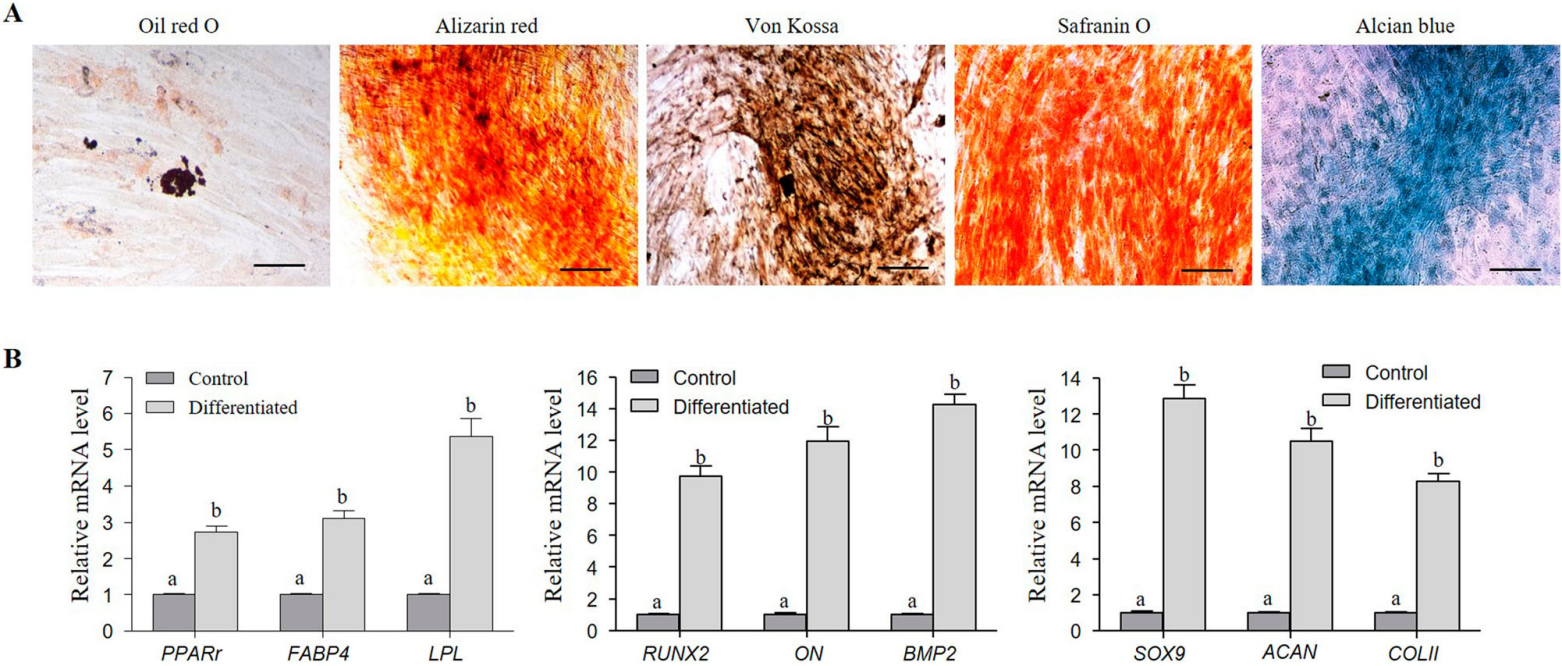
In vitro differentiation of DPSCs into mesenchymal lineages. (A) Differentiated cells were evaluated by lineage specific staining (Oil red O for adipocytes, Alizarin red and von Kossa for osteocytes, and Safranin O & Alcian blue for chondrocytes) (Scale bar = 100 µm). (B) RT-qPCR analysis of fold change in the mRNA expression of lineage-specific genes. The relative mRNA level was quantified using 2^-ΔΔCT^ method. Tyrosine 3-monooxygenase/tryptophan 5-monooxygenase activation protein, zeta polypeptide (*YWHAZ*) was used as normalization control. The expression levels are expressed as the fold change relative to the undifferentiated DPSCs [Adipocyte-specific markers: peroxisome proliferator-activated receptor gamma-2 (*PPARγ*), fatty-acid binding protein 4 (*FABP4*), and lipoprotein lipase (*LPL*); Osteocyte-specific markers: runt-related transcription factor 2 (*RUNX2*), osteonectin (*ON*), and bone morphogenetic protein 2 (*BMP2*); and chondrocyte-specific markers: sex determining region Y-box 9 (*SOX9*), cartilage-specific proteoglycan core protein (*ACAN*), and type II collagen (*COLII*)]. Data represent mean ± SEM from three independent experiments. Significant differences are denoted using different letters when *p* < 0.05.

DPSCs were analysed for their ability to differentiate into mesenchymal lineages, such as adipocytes, osteocytes, and chondrocytes under lineage-specific in vitro differentiation conditions. Successful differentiation into adipocytes was indicated by the accumulation of intracellular lipid droplets after staining with Oil red O (Figure 2(A)). The differentiated DPSCs expressed adipogenic lineage-specific marker genes such as peroxisome proliferative activated receptor gamma (*PPARγ*), fatty acid binding protein 4 (*FABP4*), and lipoprotein lipase (*LPL*). The RT-qPCR analysis indicated an upregulated expression of these markers in the differentiated DPSCs when compared to the undifferentiated control DPSCs (Figure 2(A,B)). Upon osteogenic induction, the DPSCs differentiated into osteocytes, as evinced by the formation of mineralized nodules upon staining with Alizarin red and von Kossa (Figure 2(A)). Furthermore, the differentiated DPSCs exhibited an upregulated mRNA expression of the osteogenic lineage-specific marker genes such as runt-related transcription factor-2 (*RUNX2*), *osteonectin (ON)*, and bone morphogenetic protein 2 (*BMP2*) when compared to the undifferentiated control DPSCs (Figure 2(A,B)). Upon chondrogenic induction, the DPSCs successfully differentiated into chondrocytes, as demonstrated by the deposition of glycosaminoglycan and sulphated proteoglycans upon staining with Safranin O and Alcian blue (Figure 2(A)). These differentiated DPSCs showed increased expression of the chondrogenic lineage-specific marker genes such as SRY-Box 9 (*SOX9*), *AGGRECAN (ACAN)*, and *COLLAGEN II (COLII)* compared to the undifferentiated DPSCs (Control) (Figure 2(A,B)).

### In vitro differentiation of DPSCs into cholinergic neurons

**Figure 3.**
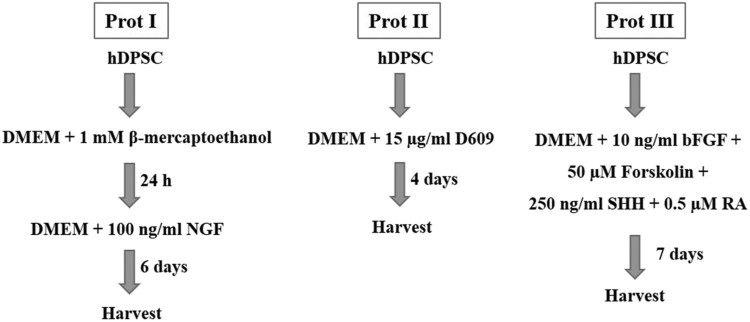
Schematic representation of the in vitro differentiation of DPSCs into cholinergic neuron-like cells using three different protocols.

**Figure 4.**
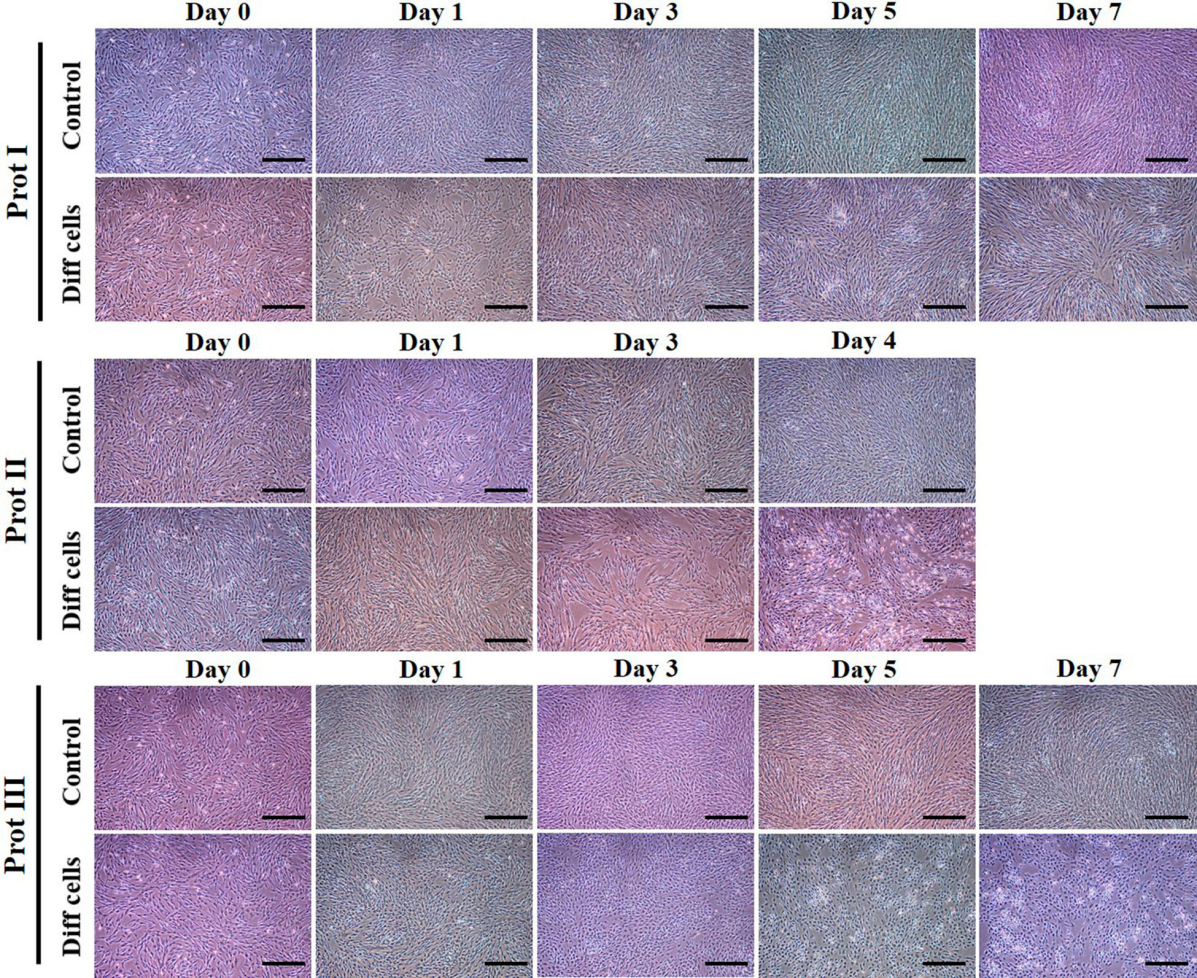
Morphological changes observed under phase contrast microscope during in vitro differentiation of DPSCs into cholinergic neurons. DPSCs were successfully differentiated into cholinergic neuron-like cells with all the three protocols evaluated in this study. Morphological changes were observed throughout the differentiation process and compared to the undifferentiated control groups. Differentiated cells from protocol I appeared more or less elongated when compared to the other two protocols. Significant morphological changes were evident in the cells obtained by following protocols II and III, where cells showed altered behaviour (Scale bar = 100 µm).

**Figure 5.**
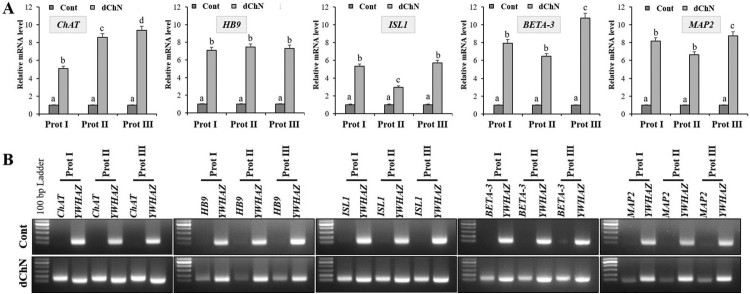
Evaluation of the expression of cholinergic neuron-related genes after differentiation. (A) RT-qPCR analysis to evaluate the fold change in the expression of cholinergic neuron-related marker genes such as *ChAT*, *HB9*, *ISL1*, *BETA-3*, and *MAP2*. The expression levels are derived from the changes in the mRNA levels relative to the undifferentiated control using 2^-ΔΔCT^ method. Tyrosine 3-monooxygenase/tryptophan 5-monooxygenase activation protein, zeta polypeptide (*YWHAZ*) was used as housekeeping control for normalization. Data represent mean ± SEM from three independent experiments. Significant differences are denoted using different letters when *p* < 0.05. (B) Agarose gel electrophoresis to evaluate the product size of each marker gene.

**Figure 6.**
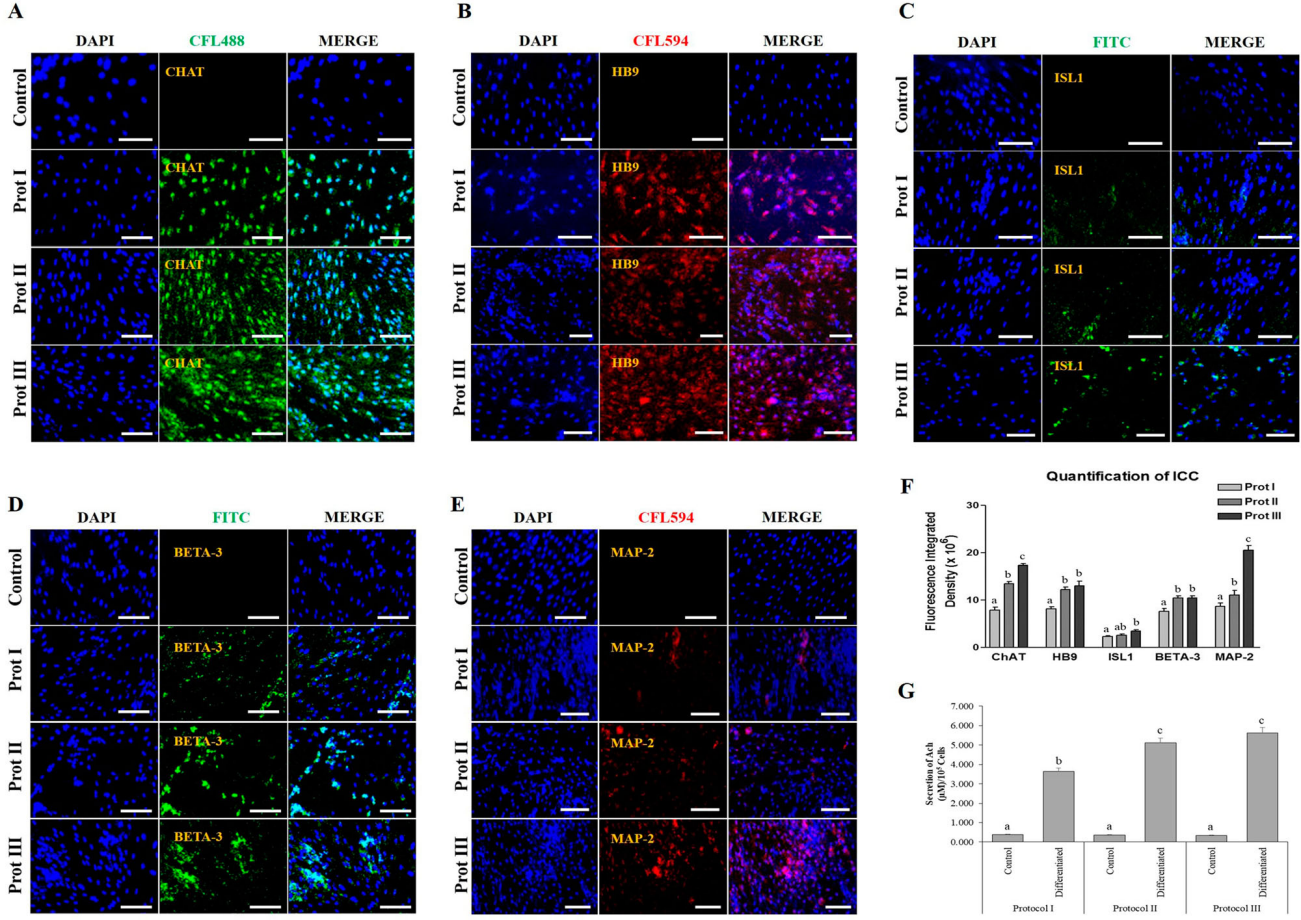
Immunocytochemical (ICC) analysis and the functional properties of in vitro differentiated cholinergic neurons. (A-E) Differentiated cells under all the three protocols strongly expressed cholinergic-specific proteins such as ChAT, HB9, ISL1, BETA-3, and MAP-2, whereas the same proteins were not expressed in undifferentiated control DPSCs (Scale bar = 100 µm). (F) Quantification analysis of ICC revealed that the differentiated cells under protocol III showed higher expression of cholinergic marker proteins compared to the other two protocols. (G) Quantification of acetylcholine (Ach) by ELISA. The level of Ach was analysed in the culture media using a biochemical fluorescent assay, indicating that all the differentiated cholinergic neurons could synthesize Ach. However, protocol III showed the highest Ach secretion. Data represent mean ± SEM from three independent experiments. Significant differences are denoted using different letters when *p* < 0.05.

In order to evaluate the transdifferentiation potential of DPSCs into cholinergic neurons, the cells were allowed to grow until they reach 70% confluence before induction. Our present study intended to compare three different protocols for in vitro differentiation, which are schematically outlined in Figure 3. The DPSCs successfully differentiated into cholinergic neuronal-like cells in all the protocols evaluated in the present study. Morphological changes were observed throughout and compared to the undifferentiated control groups (Figure 4). When protocol I was used, the resultant differentiated cells appeared less elongated when compared to the differentiated cells obtained using the other two protocols. Significant morphological changes were evident in the differentiated cells achieved using protocol II and III, where cells tended to elongate and showed altered morphology (Figure 4). RT-qPCR analysis showed a significant increase in the expression of *ChAT*, *HB9*, *ISL1*, *BETA-3*, and *MAP2* in the differentiated cholinergic neurons (dChN) compared to the undifferentiated cells. Interestingly, dChN obtained using protocol III showed significantly (*P* < 0.05) higher fold change in mRNA levels of cholinergic neuron-related genes when compared to the dChN from the other two protocols (Figure 5(A,B)). Furthermore, the resultant dChN using all three differentiation protocols showed strong expression of cholinergic-specific proteins such as ChAT, HB9, ISL1, BETA-3, and MAP-2 by immunocytochemistry, whereas these proteins were not expressed in the undifferentiated DPSCs (Figure 6(A-E)). Further, the quantification of ICC results demonstrated higher expression of the cholinergic marker proteins in the differentiated cells obtained by using protocol III compared to the cells resulted from the other two protocols (Figure 6(F)).

Ach secreted from the differentiated and undifferentiated cells was quantified using the culture supernatants. The differentiated cells obtained by using all the three induction protocols were able to secrete Ach significantly compared to their undifferentiated counterparts (Figure 6(G)). The highest concentration of Ach (µM/10^5^ cells) was observed from the differentiated cells obtained using protocol III (Figure 6(G)). Collectively, treated cells from protocols II and III not only showed more altered morphologies but also demonstrated high staining intensity for cholinergic markers and comparatively more Ach secretion. Furthermore, DPSCs induced with protocol II and III shared comparable expression levels. However, a significantly higher expression level of markers such as *ChAT and MAP2* were observed in protocol III induced DPSCs in comparison to other treatment groups. Taken together, these results suggest that both the protocols II and III could more efficiently promote the cholinergic neuron-like cell differentiation potential of DPSCs. However, protocol III induced DPSCs showed marginal higher differentiation potential.

### Discussions

In accordance with previous reports, DPSCs isolated from the dental pulp tissue exhibited fibroblast morphology upon in vitro culture. These cells expressed the pluripotent markers such as OCT4, SOX2, and NANOG both at the mRNA and protein levels, and positive for MSC-specific cell surface markers (Jang et al. 2018). Further, the DPSCs successfully differentiated into the mesenchymal lineages, such as adipocytes, osteocytes, and chondrocytes (Jang et al. 2018). Similarly, the DPSCs extracted from the wisdom teeth are multipotent stem cells possessing MSC characteristics in the present study.

Until now, various protocols have been followed for the differentiation of these stem cells into cholinergic neurons. Previously, we have successfully differentiated DPSCs to cholinergic neuron-like cells by inducing with tricyclodecane-9-yl-xanthogenate (D609), a specific inhibitor of phosphatidylcholine-specific phospholipase C (PC-PLC) (Jang et al. 2018). However, in the literature, many other chemicals, cytokines, and growth factors were used for cholinergic or motor neuron differentiation from stem cells (Wang et al. 2004, 2007; Goncalves et al. 2009; Naghdi et al. 2009a, 2009b; Qi et al. 2010). In this study, we compared the use of growth factors and cytokines such as NGF, bFGF, forskolin, SHH, and RA along with D609 for efficient differentiation of DPSCs into cholinergic neurons using three different published protocols.

Protocol I in the present study involves the addition of BME for pre-induction and NGF for the differentiation of nerve cells (Naghdi et al. 2009a, 2009b). Earlier, the use of BME as a pre-inducer for differentiation of bone marrow MSCs (BMSCs) to neurons has been reported by Woodbury et al. (Woodbury et al. 2000). BME, with strong anti-oxidant and thiol reduction potentials, induces BMSCs to express neuroblastic markers such as nestin and NF-160. On the other hand, NGF has been reported to exhibit anti-apoptotic, trophic, and differentiating functions in the sympathetic neurons (Koike and Tanaka 1991), enhance the expression of genes regulating the acetylcholine synthesis (Madziar et al. 2005), and allow the maturation and repair of the basal forebrain and striatal cholinergic neurons in vivo (Pean et al. 2000). Generally, BME and other antioxidants such as N-acetylcysteine inhibit neuronal apoptosis by increasing the glutathione levels. This increased glutathione level was further implicated in an increase in ChAT activity and alteration in the neurite outgrowth patterns of the cholinergic precursor cells of the basal forebrain (Ni et al. 2001). Although, the induced cells could show the expression of specific markers both at mRNA and protein levels but the expression level was comparatively low in comparison to other protocols used in the study. Possible reason behind these observations could be the requirement of additional supplements or differentiation promotors which could enhance the extent of differentiation at a comparable or more satisfactory level.

Protocol II in the present study is a simple method to induce the cholinergic nerves from stem cells by adding D609 to the induction media (Wang et al. 2004, 2007; Sun et al. 2013). D609 is a specific inhibitor of PC-PLC. Several reports have also demonstrated the use of D609 in differentiating the bone marrow MSCs into morphologically neuron-like cells, which are possessing cholinergic neuronal characteristics (Wang et al. 2004; Shao et al. 2012; Sun et al. 2013). Upon D609 treatment in MSCs, PC-PLC inhibition and increased levels of heat shock protein 70 (HSP70) which activates the transcription regulator B-cell translocating gene 2 (BTG2), was observed. This resulted in up-regulation of the functional neuronal specific genes and acceleration of the cholinergic neuronal differentiation (Shao et al. 2012; Jang et al. 2018). In addition, D609 treatment was shown to downregulate the genes related to mesodermal and endodermal differentiation. On the contrary, D609 upregulated the expression of the gene involved in neuronal differentiation, neuroprotection, and cholesterol synthesis (Shao et al. 2012). These observations strengthen the utility of the used protocol which seems to be efficient when DPSCs are induced under a shorter duration of time. Main importance of this protocol is its low induction period which could be more beneficial in the application to mass production of specific neurons. Protocol III used in the present study involved the use of bFGF, SHH, and RA (Goncalves et al. 2009; Qi et al. 2010). Further, forskolin was added in addition to the three factors. Forskolin was reported to induce neuronal phenotypes by expressing the immature neuronal markers but not the mature neuron markers, such as synapsin in BMSCs. However, the use of SHH and RA along with forskolin was found to significantly improve neuronal differentiation (Qi et al. 2010). The use of RA as a neuronal inducer has been reported previously (Kim et al. 2002; Jin et al. 2003). A previous study indicated that RA induces BMSCs to express mature neuron markers and an acetylcholine transmitter (Qi et al. 2010). RA is derived from vitamin A, which plays an important role in proliferation and differentiation in adult neurogenesis (Goncalves et al. 2009). SHH is a member of the hedgehog family of signalling molecules. It is a protein secreted from two signalling centres, the notochord, and the floor plate (Kondo et al. 2005). The RA signalling cascade involved in neurogenesis was found to crosstalk with other pathways such as FGF and SHH signalling (Ribes et al. 2005). Previous studies demonstrated that the FGF signalling pathway promotes neurogenesis in the developmental stage and drives the neuronal differentiation in neuronal precursor cells (Kosaka et al. 2006; Goncalves et al. 2009). In addition, SHH signalling has been linked to neurogenesis in the adult brain (Palma et al. 2005).

In the present study, DPSCs isolated using all the induction protocols successfully differentiated into cholinergic-neuron like cells. However, few differences were noticed in the degree of differentiation and the expression of neuronal markers between the cells obtained from three different induction protocols. DPSCs isolated using protocol I showed the lowest cholinergic differentiation potential and lower cholinergic markers expression and Ach secretion compared to cells obtained using the other two protocols. Further, the cholinergic neuronal differentiation with D609 (protocol II) resulted in an increased expression of cholinergic neuron-related markers and Ach secretion compared to that in cells obtained using protocol I. Though the exact mechanism behind this difference is not clear, we speculate from previous studies that the production of reactive oxygen species (ROS) as a response to NGF treatment in protocol I might have caused the reduction in the neurogenic differentiation potential (Suzukawa et al. 2000). D609 treatment in protocol II might have decreased the generation of ROS to prevent the mature neurons from entering the cell cycle to benefit the neuroprotection (Adibhatla and Hatcher 2010). However, in the present study, Ach concentration and most of the cholinergic neuronal markers were highly expressed in the differentiated cholinergic neurons obtained following protocol II and III. While protocol III induced DPSCs were shown to have marginal higher expression both at mRNA and protein level. This observation indicates that the combination of bFGF, SHH, and RA in the differentiation protocol not only increased the expression of neuronal markers but also increased the secretion of neurotransmitters. Similar to our current study, a previous report indicated an increased cholinergic neuronal differentiation of bone marrow-derived MSCs using these factors compared to the use of a single biochemical factor (Qi et al. 2010). Moreover, there exists an interplay between the bFGF, SHH, and RA signalling, which was implicated in neurogenesis (Goncalves et al. 2009).

## Conclusions

Although further studies including electrophysiological functional tests are needed, the present study demonstrated a choice able approach to differentiate MSCs into cholinergic neuron-like cells from human dental pulp stem cells when either cultured with D609 or bFGF, SHH, and RA in the media. DPSCs induced under different protocols were shown to have varied but positive differentiation ability. Our observations from the current study provide potential insights into its implications in therapeutic medicine, especially in the fields of motor and cholinergic nerve regeneration.
